# Preliminary Adaptation, Development, and Testing of a Team Sports Model to Improve Briefing and Debriefing in Neonatal Resuscitation

**DOI:** 10.1097/pq9.0000000000000228

**Published:** 2020-01-27

**Authors:** Rebecca Jordache, Cora Doherty, Celyn Kenny, Paul Bowie

**Affiliations:** From the * Cardiff University Medical School, Cardiff, UK; † University Hospital of Wales, Neonatal Intensive Care Unit; ‡ University Hospital of Wales, Neonatal Intensive Care Unit; § Institute of Health and Wellbeing, University of Glasgow, Glasgow, UK.

## Abstract

Briefing in team sports has been shown to benefit both performance and confidence among team members. The neonatal resuscitation team shares similarities with sports teams, where task performance includes rapid decision-making skills within dynamic situations, alongside unpredictable circumstances.

**Aim:**

We aimed to determine the effect of a team sports briefing model on the neonatal resuscitation team. Method: We adapted and redesigned a team sports briefing and debriefing model and related protocol and tested them with a neonatal resuscitation team in a U.K. university teaching hospital.

**Results:**

The team’s confidence and perceptions around these 2 aspects of resuscitation were studied along with frequency of task execution before and after a teaching intervention about the sports teams' approach to briefing and the introduction of a 7-point checklist. In 20 preintervention observations of 13 key tasks, areas for improvement in how neonatal intensive care unit (NICU) staff prepared for deliveries and potential resuscitations were found such as in “discussing potential deliveries that day” (n = 10, 50%), “identifying roles within the resus team that shift” (n = 5, 20%), and announcing when and where a debrief would occur (n = 0, 0%). Postintervention, the NICU team’s mean task completion increased significantly from 9.23 (SD = 6.34) to 18.0 (SD = 1.83), a statistically meaningful difference of 8.77 (95% CI, 4.99−12.55; *P* < 0.001).

**Conclusion:**

Together with increased levels of confidence and efficiency reported postintervention, this provides some evidence that a brief–debrief process based on how sports team’s approach pregame situations could be potentially transferable and beneficial to NICU team performance.

## Abstract 2

Supplemental Digital Content is available in the text.

## INTRODUCTION

Neonatal resuscitation aims to establish sufficient spontaneous breathing and cardiac output to prevent morbidity and mortality associated with hypoxic–ischemic tissue injury.^1^ The transition from the intrauterine to extrauterine environment requires intervention by a skilled team in 10% of deliveries, with 1% of deliveries requiring extensive resuscitation measures. Extreme prematurity is the main complication of pregnancy that requires complex resuscitation with 80% of low birth weight infants requiring resuscitation and stabilization at delivery.^2^

Clinical management during neonatal resuscitation is important and can influence long-term outcome.^3^ The attending resuscitation team need to deliver resuscitation and adapt to the physiological problems the infant is experiencing. This requires rapid assessment skills, a good understanding of the physiology, an effective way of communicating between the team, and efficient execution of the list of technical skills required.^2^ Although practice in industry has often been applied to medicine,^3^ we felt the skills used in resuscitation may be more applicable to the skills and task execution employed by sports teams during a game. Moreover, there is some evidence^4^ that there is value in comparing success for sports teams and healthcare teams, with many skills and related processes potentially transferable to the health professional working environment.^5,6^

In addition to the tactics employed on the pitch, sports teams carry out well-established pre- and postgame briefings which elite sports players feel are invaluable for achieving team success^7^ (R.J. plays for the England Lacrosse team). It is felt that these practices help in enabling team members to initiate communication early in the task process, facilitating the assignment of team roles, ensuring adherence to established protocols, flagging potential problems that may arise, and standardizing a process for ensuring effective execution (R. Jordache, personal communication, April 2018). In sport, it would be inconceivable that a prematch briefing would not occur; the quest for improved safety and quality would suggest that the same level of behavior should be embedded in healthcare. However, the format of any briefing needs to empower and raise confidence to be fully appreciated and embedded in routine clinical practice.

The Neonatal Resuscitation Council now recommend the use of briefing and debriefing processes as a safety measure around resuscitation.^8,9^ Although they state that the process involves reviewing and communicating pertinent facts about the resuscitation before and after events,^9,10^ a clear framework that can be implement and embedded into practice has not been provided.

We therefore hypothesized that a well-established sports Briefing Model may be beneficial and applicable to Neonatal Resuscitation. We aimed to (1) adapt, redesign, and implement a Team Sports Briefing and Debriefing Model for neonatal resuscitation and (2) determine if there was a measurable improvement in task execution and confidence perception of the team over two 5-day periods, both before and after the introduction of the model.

## METHODS

### Setting and Participants

This study was undertaken at the regional tertiary neonatal intensive care unit (NICU) in a large university teaching hospital in Wales. There are 28 baby cots and over 550 admissions annually with a delivery rate of 6,200. The NICU is staffed by a multidisciplinary team of Consultants at tier 3 (n = 10), tiers 1 and 2 Advanced Neonatal Nurse Practitioners and Junior Doctors (n = 20), and various grades of nurses. Members of the whole team actively participate in neonatal resuscitation with the team varying from shift to shift (minimum shift duration being 8 hours). Team members have Neonatal Life Support training.

### Cross-sectional Questionnaire Survey

An initial baseline short questionnaire survey (designed by R.J. and C.D.) was distributed to a number of members of the resuscitation team (25 doctors and 35 nurses) with the aim to assess the neonatal team’s perceptions of, and confidence in, the current in-house briefing and debriefing process, in addition to the effects of task execution during resuscitation.

### Adaptation and Redesign of the Team Sports Briefing–Debriefing Model

The Briefing–Debriefing Model (BDM) routinely applied by the Senior Women’s England Lacrosse Team was reviewed by the team, and this particular model was chosen as R.J. had frequent practical pitch side experience of that model. The model contains 13 essential tasks. These tasks are applied before a match (the Briefing Model) and then following a match (the Debriefing Model). Tasks on the model were studied to determine which ones might be applicable to steps in neonatal resuscitation (Appendix 1, available at http://links.lww.com/PQ9/A138). Relating briefing tasks between a lacrosse game and a resuscitation could be easily identified and included steps such as equipment preparation, self preparation, and role identification in the Briefing task set. Standard steps in the Debriefing task set included the leader summary and team involvement. To allow for practical applicability for the on call neonatal resuscitation team, these briefing tasks were then modified and renamed Briefing Model 1 (R.J., C.D., C.K.) (Appendix 2, available at http://links.lww.com/PQ9/A139). The aim was then to review the level of baseline tasks being undertaken by the resuscitation team before any intervention or training about Sports Teams Briefing.

### Preintervention Use of Briefing Model 1

The agreed briefing model essential tasks (Appendix 2, available at http://links.lww.com/PQ9/A139) were used by R.J. when observing 20 deliveries that the resuscitation team were called to over a 5-day period. Using Briefing Model 1 as a checklist, observational data were collected on the completion of specific tasks at the beginning of the shift (eg, Preparing the resus team and identifying roles within the team) or when a resuscitation call occurred. A percentage of successful task execution was generated.

### Intervention

The principles and methods underpinning the concept of briefing/debriefing and the preliminarily developed BDM document were introduced to NICU clinical staff (n = 34) at an interactive session led by R.J. during April 2017, which outlined the purpose of the Team England Lacrosse’s BDM and its perceived potential transferability and benefits to the NICU. The development of Briefing Model 1 was reviewed and discussed between project leads and NICU staff during the teaching session and resulted in mutual agreement that, for ease of use and applicability, the 13 essential tasks could be condensed to a 7 task briefing checklist or bundle, to be known, for the purposes of this article, as Briefing Model 2 (Box 1, available as Supplemental Digital Content at http://links.lww.com/PQ9/A145). The items in the updated 7-point checklist would still permit a postintervention assessment using Briefing Model 1 as an observation checklist. A training program to implement the use of Briefing Model 2 was then undertaken. Regular daily teaching sessions took place with the wider team, champions were engaged, and information sheets were placed at work stations and on notice boards around the NICU.

### Postintervention Use of Briefing Model 1

Following the training program, R.J. observed a further 20 deliveries that the resuscitation team were called to over another 5-day period. The same redesigned Briefing Model (Appendix 2, available at http://links.lww.com/PQ9/A139) that was used before the intervention was then again used as a checklist, ensuring continuity and standardization. To determine if observed task performance improved by chance or otherwise, differences in proportions of and mean task execution pre- and postintervention were calculated along with 95% confidence intervals and a probability value of significance using a Chi-square test.

### Postintervention Questionnaire Survey

Following the intervention, a shorter more specific questionnaire was sent out to determine if the team believed the training and checklist had/would increase their confidence when going to a delivery and/or resuscitation. Questions were carefully worded to allow for comments regarding negative consequences of the intervention to be recorded in free-text form.

## RESULTS

### Survey Questionnaire Findings (Preintervention)

**Figure 1.**
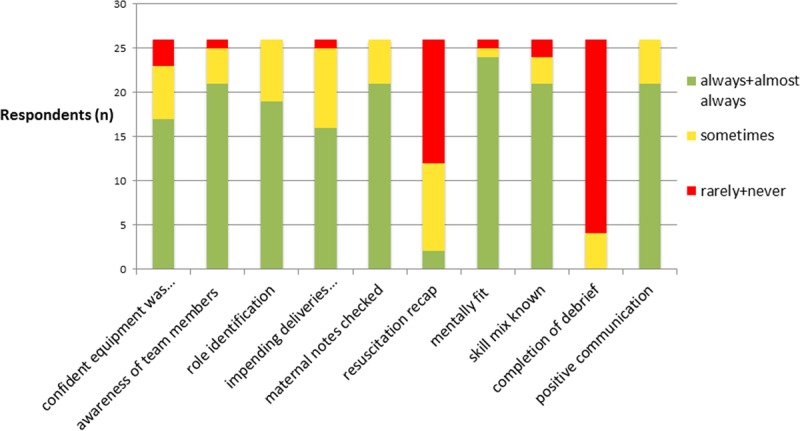
A stacked bar chart to show the NICU staff’s responses from the first questionnaire (n = 26). These questions are in relation to how confident they felt that each task was executed before the intervention.

A total of 26 team members completed the survey equating to a response rate of 43.3% (11/25 doctors; 44.0%; and 15/35 nurses, 43.0%). The most positive feedback about the current briefing and debriefing practice was reported on confidence that the team attending a delivery communicates effectively and positively (20/26, 77.0%); other important issues included 13 of 26 (50%) respondents reported that the stages of resuscitation are rarely or never recapped with the team before a delivery, whereas 22 of 26 (85%) indicated that a debrief rarely or never takes place following a delivery. The main results are outlined in Figure 1.

### Survey Questionnaire Findings (Postintervention)

The postintervention questionnaire (Appendix 3, available at http://links.lww.com/PQ9/A140) was sent out to 25 doctors and 35 nurses on the unit after the intervention, and there was a 20% response rate (n = 12), from 7 doctors and 5 nurses. Although this was a low response rate, the main findings regarding how staff felt show that the checklist points were useful to address at the start of a shift, that it was realistic to set aside time to do so, and that the checklist points helped improved communication across the team when called to a delivery and increased the team’s confidence when attending a delivery. Combining all responses, 93.8% of responses (n = 50) were in the “definitely yes” or “yes” categories on the Likert-type scale the majority of the time. It was the “Do you think ensuring a short debrief took place after each delivery will help improve teamwork and efficiency in the long run?” question that held more uncertainty with these being the responses: 4 answering “definitely yes,” 4 answering “yes,” the majority of the time, and 4 answering “maybe.”

### Results from the Preintervention Testing of the Briefing Model 1

Observations from the Briefing Model 1 that occurred before the shift and before deliveries showed particular patterns (Table 1). From the initial task observations, after adapting the Briefing Model 1, tasks such as “discussing potential deliveries that day” only occurred in 50% (n = 10/20) of cases (n = 4/20), “identifying roles within the resus team that shift” happened in 20% of cases (n = 20), and announcing when and where a debrief would occur happened in 0% of cases. Overall, from the 13 tasks that were being observed in 20 different calls to the delivery room, a total of 46.2% (n = 120/260) of the tasks were completed.

**Table 1.**
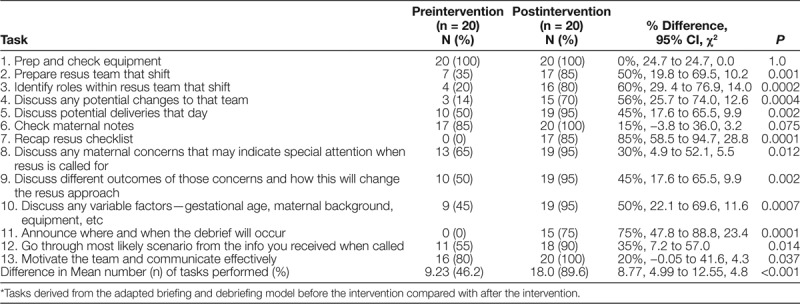
Pre and Postintervention Observed Frequency of Task Execution* in 20 Deliveries with Calculated Differences in Proportion, 95% Confidence Intervals and Chi-Square Tests

### Results from the Postintervention Testing of the Briefing Model 1

The postintervention observations (Table 1) show a significant increase in the total frequency of the tasks carried out, from 46.2% (n = 120/260) to 89.6% (n = 233/260). The task of “announcing when and where the debrief will occur” was completed 75% (n = 15/20) of the time, showing the largest improvement as this figure was initially 0%. The second most improved task by measure of frequency was “identifying roles within resus team that shift,” which increased by 60%. The mean total task execution was statistically significant (*P* < 0.001) indicating that the observed improvements were highly likely due to the success of the intervention rather than by chance (Table 1).

## DISCUSSION

Briefing has been used in sport and has been shown to be positively associated with success. The neonatal team rely on good communication; however, using a sports performance improvement method was worth assessing in NICU as we recognized the similarities in task execution between sports games and neonatal resuscitation/initial stabilization activities. Due to personal experience, the BDM of the England Lacrosse team was adapted to the neonatal resuscitation environment and pilot tested. The findings suggest that developing a training program and then implementing a simple checklist or bundle helped to facilitate improvement in perceived confidence levels and task execution by the NICU team. However, this is unsustainable without someone championing the project. The variation in task execution alone before the intervention shows that some principles are very embedded in culture, whereas others could be improved. Before the intervention, variation in task execution was apparent particularly in relation to when and where a debrief may occur. At that time, the unit had a well-established practice of holding informal unstructured debriefs immediately following a neonatal death, followed by a more formal debrief at a planned time and location for all members of the neonatal team, with wider team members such as midwives and obstetricians also being invited. The informal debriefs were often ad hoc, whereas the formal debriefs were associated with very positive feedback when assessed (R. Jordache and C. Doherty, personal communication, May 2017, from another quality improvement process on the NICU). In the current project, a debrief did not occur during the preintervention period in any of the observed resuscitation episodes. These results are not surprising as none of the resuscitations were extensive or involved a death. Following the intervention, however, the debrief task execution in observed deliveries increased to 75%. This was a positive change and is supported by the Neonatal Resuscitation Program (NRP) recommendation that “briefing and debriefing techniques be used whenever possible for neonatal resuscitation.”^11^ In addition, the importance of a debrief is becoming increasingly recognized within neonatal care and has been recently incorporated into quality frameworks,^12^ in addition to being regarded an important part of the resuscitation process by other teams.^13^ Moreover, England lacrosse have long recognized the benefit of a brief and debrief. These processes are now embedded in sports culture, and it would now be inconceivable for a brief or debrief to not happen, a scenario that is still some way off in routine neonatal pediatric practice in most health systems.

The briefing/debriefing model and simplified checklist used in this project encouraged the staff to address tasks to ensure they were carried out in the first place and managed accordingly. The increase in task frequency after the intervention provides evidence of this. Through the adapted 7-point checklist, the team were reminding each other and themselves to carry out the tasks deemed necessary from the adapted BDM.

The feedback from the survey was positive with additional comments highlighting that developments could be made to further improve the briefing checklist. It is challenging, however, to determine whether improved team confidence and efficiency improved the task execution, or whether an increase in task execution resulted in improved confidence and efficiency. Either way, the survey reflects an acceptance of the checklist in relation to improving clinical practice.

For this intervention to continue, it is important to have a team to champion the project; however, it has been shown that within a short time frame with adequate teaching and a commitment to improvement, a briefing protocol can be readily accepted. This pilot study acted as a platform for the introduction of a team huddle to clarify who the members of the resuscitation team are. This is now embedded in practice at the start and end of each day on the NICU.

The observation from this study is that new ideas and processes are readily accepted; however, sustaining and embedding them into practice requires continued effort as with any quality improvement measure.^14^ Incorporating the checklist into any framework for briefing and debriefing may help to embed a culture change needed to truly advance safety and quality. Although the tertiary neonatal unit has already applied a number of sports analogies into practice, this is the first example that we are aware of where an established sports briefing and debriefing model was used in neonatal care. The outcomes of this study help support the existing evidence of using briefing and debriefing in neonatal resuscitation,^13^ but it is hoped that this may inspire and drive other projects in medicine toward safer and more effective briefing and debriefing practices, based around a sports team model.

### Study Strengths and Limitations

The BDM has strong face-validity, and this study shows that the 7-point checklist if adopted by the NICU team could potentially enhance confidence perception and task execution. However, to accurately measure sustainability, observations would need to be performed a number of times per week or month to monitor improvement progress over time. This would allow more robust statistical analysis of the intended improvement, which is core to quality improvement science and practice.

It is also acknowledged that the postintervention survey response rate (20%) is low compared with the average return rate (43.3%) for the preintervention survey. It is recognized that maintaining full time presence of champions on the NICU may have promoted greater survey completion.

The scope of the project did not allow outcome data (neonatal survival/harm) to be collected. It is therefore not possible to claim that greater task execution is associated with improved outcomes; however, one could assume that greater task execution may reflect improved team communication which has been shown to be associated with improved clinical outcomes. Sauer et al^15^ reported improvements in neonatal outcomes via the promotion of teamwork and communication between the obstetrician, labor and delivery staff, and the neonatal resuscitation team by implementing a team prebrief, debrief, and delivery room checklist. The initial task observations and questionnaires compared with the postintervention task observations and confidence levels from the team suggest that the Briefing Model and checklist helped to improve motivational and efficient communication. This has also been seen in surgical specialties, such as in Jain et al^16^ where surgeon satisfaction increased along with fewer delays and interruptions (ie, increased efficiency) when a preoperative huddle was introduced before each operating case. As stated above, the team huddle has now been introduced to the NICU following this pilot and is well accepted.

## CONCLUSIONS

Healthcare is continually striving to improve system performance and the wellbeing of people. This small pilot project demonstrated that the potential for improvement in NICU staff performance may be achievable by learning from professional sport teams which have significant experience in implementing and mastering a briefing and debriefing structure to perform at their best, although more rigorous development, implementation, and testing is recommended to determine the true benefit.

## ACKNOWLEDGMENTS

Ethical review is not required for service evaluation, developments, or quality improvement projects in the United Kingdom.

## Supplementary Material


