# Supplementary material for: Preliminary Adaptation, Development, and Testing of a Team Sports Model to Improve Briefing and Debriefing in Neonatal Resuscitation
Source: Pediatr Qual Saf. 2020 Jan 27;5(1):e228. doi: 10.1097/pq9.0000000000000228 (PMC7056292; doi:10.1097/pq9.0000000000000228)
Supplement: Supplementary file 1 [file pqs-5-e228-s001.docx]

**Appendix 1** – The initial briefing and debriefing model. - description and comparison of the briefing and debriefing processes between England Lacrosse and Neonatal Resuscitation

| **BRIEFING** | **ENGLAND LACROSSE** | **NEONATAL RESUSCITATION** |
| --- | --- | --- |
| Equipment Preparation | Prep equipment - group (ball bag, cones, first aid kit) and individual (stick, mouth guard, pinnie, boots, water bottle, electrolyte tablets, recovery shake). | Individual equipment (scrubs, personal hygiene, stethoscope) and team equipment (suction machine, oxygen mask, resuscitative sheets, incubator). |
| Self Preparation | Prepare own self for play - physio, hydration, nutrition | Prepare resus team that shift – ensure everyone has carried out the NLS course and then ensure the bleep is handed over to the right person, and if more than 1 person is needed to attend a resus, that the individuals involved are notified before. |
| Role identification | Coach/captain will read out the team sheet and starting line up, including positions. | Identify roles within the resus team that shift - ensure the leader of the group is established, and roles for each member is known when it comes to assisting in the resus – ventilation, compression, medications etc. |
| Team alterations | Rotations if substitutes are needed – discuss how having different people on at a time may effect what pre planned moves you can do | Discuss any potential changes to that team – discussion with team at the start of the shift - if a call happens at a certain time in the day, will members always be around, if one can’t be found, is there a plan for someone to go instead? |
| Attacking focus | Is it something we need to practice, or something the opposition is going to challenge us on, or something we need to execute well? | Discuss potential deliveries that day - know how many expectant mothers there are on the delivery suite, know of any elected c-sections, especially in those of pre-term babies. (This is proactive, with the upper hand, the first move, much like the attacking unit in a team). |
| Defensive focus | Is it something we need to practice, or something the opposition is going to challenge us on, or something we need to execute well? | Discuss the response to any unexpected resuscitations - (This is reactive rather than proactive, much like the defending unit in a team). And check the maternal notes for any potential challenges that may arise |
| Recap | Recap moves/plays – go over set play and ensure everyone is on the same page and knows what their job personally is. | Recap NLS resus checklist - talk through the steps that may be needed if the baby needs resuscitating. |
| Indirect threats | Discuss opposition and how this may change our game plan - they may have a certain playing style that could affect the way we like to attack so we will have to adapt. | Discuss any maternal concerns in notes that may indicate special attention when resus is called for - eg pre-eclampsia, sepsis, hypertension, gestational diabetes, smoking/alcohol |
| Direct threats | Prepare for any potential threats to our team and how we will deal with them - they may have 1 very talented player that may be hugely influential in their goal scoring. | Discuss different outcomes of those concerns and how this will change the resus approach. |
| Variable factors | Discuss any variable factors – surface of pitch, weather, quality of umpires, team fatigue | Discuss any variable factors – gestational age, maternal background, equipment, team members strengths and expertise, mental state for appropriate decision making - if one member is much better at intubating then ensure that’s their role, if one member is mentally distracted for whatever reason, ensure they are helped and maybe not in a crucial role. |
| Debrief announcement | Announce where and when debrief will occur - we always meet back in the changing room after for a short debrief and celebration/time to be down. But we will clarify when the full team debrief will occur, probably at training the next day, allowing the team to reflect on the performance. | Announce where and when the debrief will occur – to ensure the team can attend and discuss. |
| Visualisation | Visualisation chat - the coach or captain will give us time to sit down and think about how the game is going to go and give us focus areas to think about, imagine and prep for. | Go through most likely scenario from the information you received when called - to mentally prepare for what you think may happen at the delivery and imagine what you may have to do. |
| Motivation | Motivational chat - the last thing to occur before stepping out for our warm up, to bring the team together and gear them up for the task ahead. | Motivate the team and communicate effectively - need to get everyone on the same page and ready for the delivery, and communicate well between the team to achieve success. |

| **DEBRIEFING** | **ENGLAND LACROSSE** | **NEONATAL RESUSCITATION** |
| --- | --- | --- |
| Time | Time for celebration/grief over success/loss. | Time for celebration/grief over success/failure. |
| Leader summary | Leader – coach/captain to summarise the game and outcome - this is done as an overview of what happened and what areas can be pointed out as flaws/executed well. | Leader to summarise the events – give an overview of what occurred. |
| Success/failure discussed | Reasons stated for success/failure - if there were any clear cut reasons as to why we won e.g our attacking stats showed a 90% shot conversion compared to the other team’s 45%, resulting in more successful goals. | Reasons stated for success/failure - whether anything in particular was the key aspect. And if the reason is unknown with regards to failure, then a more in depth evaluation of all resus details, maternal notes and team performance needs to be carried out. |
| Team involvement | Ask the team on their views – give a negative and positive – we usually go round and all have an input to ensure we’ve covered all areas of the pitch by asking all players, sometimes different things are picked up that the coach/captain may not have thought of/overlooked. | The debrief is opened to the resus team to discuss and their views encouraged - good to get feedback from all team members, especially as everyone was responsible for a different aspect of the resuscitation. Gives the chance for members to speak up and created a culture for this to be encouraged rather than looked down on, or seen as challenging a senior. |
| Team performance review | Team performance review - either in a feedback form or vocally, everyone discuss’ how we performed as a squad, not necessarily referring to the outcome of the game, but how we executed certain skills and planned moves/plays. | Team performance review - how did the team work in the situation and were they efficient? |
| Individual Performance review | Individual performance review - this is usually done in a feedback form so players have a chance to go away and reflect on their own performance that game and document it – so any improvements can be seen throughout the season. | Individual performance review - did you execute your task well enough and did you contribute to a good team dynamic? |
| Future training goals | Goals to focus on in training - whatever areas were picked out as being a weakness will be worked on in the next training session. Equally, anything that was executed well will also be practiced to keep the skill at a high level for increasing consistency of successful execution. | Goals to focus on in training/simulation scenarios – the action = staff leader can discuss with the team to see if there’s a certain theme in areas that members feel need practice. May need reviewing/re-training |
| Future event goals | Goals to focus on in the next game - lessons learnt from previous mistakes that will then be including in the briefing focuses of the next game. | Goals to focus on in the next call to a delivery - e.g if the maternal notes weren’t checked, ensure this is emphasised in the briefing and done next time. |
| Action plan | Plan of how to achieve those goals – what needs to be practiced more/changed - careful planning of training sessions to ensure the goals to focus on in training are done. Other areas may just need a chat and clarification between team members to be able to achieve these goals. It may mean an adjustment to the training programme. | Discuss a plan of how to achieve these goals – what needs to be done/practiced more/changed - does the leader need to organise a refresher course in challenging ventilation? |
| Recovery phase and next course of action | Appropriate recovery and rehab plan in place - each player knows what they need to do to recover and allow their body to get back to performing standard, time needs to be allowed for this, this may mean a light session/tactical team meeting instead of a physical session is on the programme for the next day. | Appropriate plan of next course of action – ITU/back to mum/parental support and trainee support. - eg. If the baby has been brought down to NICU, who is now in charge of the baby’s care, who is going to talk to the parents to keep them in the loop and what medical intervention is required. Do the attending team need a break? Any counselling? |
