# Supplementary material for: Preliminary Adaptation, Development, and Testing of a Team Sports Model to Improve Briefing and Debriefing in Neonatal Resuscitation
Source: Pediatr Qual Saf. 2020 Jan 27;5(1):e228. doi: 10.1097/pq9.0000000000000228 (PMC7056292; doi:10.1097/pq9.0000000000000228)
Supplement: Supplementary file 2 [file pqs-5-e228-s002.docx]

**Appendix 2 *–*** *Observational Tool -* Briefing and Debriefing Essential Tasks assessed Pre and Post the training and educational interventions

1. Prep and check equipment
2. Prepare resus team that shift
3. Identify roles within resus team that shift
4. Discuss any potential changes to that team
5. Discuss potential deliveries that day
6. Check maternal notes
7. Recap resus checklists
8. Discuss any maternal concerns that may indicate special attention when resus is called for
9. Discuss different outcomes of these concerns and how this will change the resus approach
10. Discuss any variable factors – gestational age, maternal background, equipment, team members strengths and expertise, mental state for appropriate decision making
11. Announce where and when the debrief will occur
12. Go through the most likely scenario from the info you received when called
13. Motivate the team and communicate effectively
