# Supplementary material for: Preliminary Adaptation, Development, and Testing of a Team Sports Model to Improve Briefing and Debriefing in Neonatal Resuscitation
Source: Pediatr Qual Saf. 2020 Jan 27;5(1):e228. doi: 10.1097/pq9.0000000000000228 (PMC7056292; doi:10.1097/pq9.0000000000000228)
Supplement: Supplementary file 3 [file pqs-5-e228-s003.docx]

**Appendix 3. A table to show the questions from the survey that was circulated post intervention, specifically about the use of the simplified BDM protocol**

| **Question** | **Results** | **Comments** |
| --- | --- | --- |
| Do you feel the first 2 points in the checklist are useful to address in a team huddle at the start of a shift? | Definitely yes = 9/12  Yes, the majority of the time = 3/12 | “Particularly for us nurses, it’s useful to know of any potential deliveries that day as usually only the doctors discuss this between them, and I may be the one who is then allocated that baby to look after if they come down to NICU, I liked being included in this conversation at the start of my shift”. - *Anonymous Staff Nurse* |
| Did you think it was realistic that the NICU team would be able to set aside a few minutes at the start of each shift to come together to address these 2 points? Was this possible at the beginning of most shifts? | Definitely Yes = 4/12  Yes, the majority of the time = 8/12 | ‘The main problem here is that nurses and doctors handover at different times as our shifts start at different times, so it wasn’t always realistic to do this part of the protocol together, nevertheless I still found it useful for both teams to address these points even if it was in 2 separate groups’. – *Anonymous SHO* |
| Do you think the next 5 points in the checklist, for when you are actually called to a delivery, did helped to improve communication across the team? | Definitely Yes = 6/12  Yes, the majority of the time = 6/12. | The aim of these 5 points was just to get the team talking and planning ahead as they attended a delivery. Comparing this to the initial survey where nearly 25% of the team only ‘sometimes’ felt confident that the team had communicated well, this shows a significant increase in effective communication |
| Do you think going over these 5 areas increased the team's confidence when attending a delivery that could potentially develop into a resuscitation? | Definitely Yes = 5/12  Yes, the majority of the time = 4/12 Sometimes = 3/12 | These results would suggest that although the content and amount of communication may have increased, this doesn’t always correlate with staff’s confidence increasing. Further investigations on how to boost confidence need to be carried out so that the points on the checklist can be more specific. |
| Do you think ensuring a short debrief took place after each delivery will help improve teamwork and efficiency in the long run? | Definitely Yes = 4/12  Yes the majority of the time = 4/12  Maybe = 4/12 | The main comments from this section were about how the team may have an informal chat about how the delivery went, but a lot of the time full resuscitation is not necessary so there’s not much to reflect on.  ‘I feel people would start to resent having to debrief after every delivery as there are so many but I definitely think a debrief would be useful after every resuscitation, we did 2 last week, after the first one I feel that the team went away feeling more proud than usual of the good job we had done after singing our praises, and after the second one, we addressed some issue that for me personally will mean I deliver better care next time’ – *Anonymous Registrar* |
