# Supplementary material for: Preliminary Adaptation, Development, and Testing of a Team Sports Model to Improve Briefing and Debriefing in Neonatal Resuscitation
Source: Pediatr Qual Saf. 2020 Jan 27;5(1):e228. doi: 10.1097/pq9.0000000000000228 (PMC7056292; doi:10.1097/pq9.0000000000000228)
Supplement: Supplementary file 4 [file pqs-5-e228-s004.docx]

***Box 1. Briefing Model 2 - The 7-point delivery and resuscitation briefing protocol***

| AT THE START OF THE SHIFT HAVE YOU: | WHEN YOU GET CALLED TO A DELIVERY: |
| --- | --- |
| 1. Identified who has the bleep, the full resus team and distrusted roles? | 3. Ensure the necessary team is attending (may be 1 member to start with) and make any necessary changes to that team |
| 2. Discussed any potential deliveries that day | 4. Prep and check all the resuscitaire equipment |
|  | 5. Recap the resuscitation stages with the team |
|  | 6. Check the maternal notes and pick out any concerns that may cause complications for the baby |
|  | 7. Decide where and when a debrief of the delivery will occur |
